# Supplementary material for: Multilocus genotyping of Giardia duodenalis in pre-weaned calves with diarrhea in the Republic of Korea
Source: PLoS One. 2023 Jan 13;18(1):e0279533. doi: 10.1371/journal.pone.0279533 (PMC9838842; doi:10.1371/journal.pone.0279533)
Supplement: Attachment — Submitted filename: Response to Reviewers.docx [file pone.0279533.s001.docx]

**We thank the reviewers for their careful reading and critical insights. These constructive comments were valuable and helpful for revising and improving our manuscript. We have provided detailed responses to the points raised by the reviewers and have modified our manuscript accordingly. The revised portions have been highlighted in red in the revised manuscript**. **We hope that the** **revised manuscript will be suitable for publication in PLoS One**

**.**

Reviewer #1: The current manuscript: Multilocus genotyping of Giardia duodenalis in calves with diarrhea in the Republic of Korea is well written and of interest. Meanwhile there are major points should be addressed.

1- Tables 1-4 were not included in the current version.

**Response: We are sorry about that. Now, we have provided all tables in the manuscript.**

2- Please, include phylogenetic trees for the obtained sequences and discuss the obtained findings.

**Response: We agree with the reviewer’s comment. We have provided the phylogenetic trees. Please see figures. We have addressed the obtained findings in the discussion. Please see lines 293-303.**

3- Author contributions, the revised version was not mentioned. All authors should approve the submitted version.

**Response: All authors have approved the final version. We have mentioned regarding this. Please see lines 354-355.**

4- Double check on the cited references.

**Response: We agree with the reviewer’s comment. We made several mistakes for citation. We have provided all citations properly.**

Reviewer #2: In this study entitled “Multilocus genotyping of Giardia duodenalis in calves with diarrhea in the Republic of Korea”, the authors investigated the infection of Giardia duodenalis in calves in ROK., and sequencing analyses showed that they belonged to zoonotic assemblage A. It was a meaningful survey of Giardia duodenalis in calves, but there were some questions.

Some questions:
1. I thought the title was vague and could be modified to better convey the message of the manuscript. Perhaps it would be appropriate to add "pre-weaned" to the title. For calves include pre weaned calves and post weaned calves.

**Response:** **We have added in response to the reviewer’s comment. Please see the title.**

2. Abstract: lines 22-25, “…causes diarrhea…”. The language of the paper should be concise, and the authors should avoid repeatitive description.

**Response: We agree with the reviewer’s comment. We have revised these sentences in response to the reviewer’s comment. Please see lines 14-16.**

3. Introduction.
Based on the information about the Giardia duodenalis provided by the authors (also include the results of this article), I learned that Giardia duodenalis is an opportunistic parasitic. But in lines 51-53 (Giardia duodenalis is a …in extreme cases) and lines 63-64(Giardia duodenalis is a … industry), the author vaguely described that as long as the host is infected with Giardia, it can have symptoms. Whether I understand it incorrectly?

**Response: We agree with the reviewer’s comment. We are sorry for the confusion. *Giardia duodenalis* infection can cause mild to severe symptoms in various hosts according to the papers we cited. In addition, *Giardia duodenalis* acts as a causative agent of diarrhea in calves. Please see lines 44-45 and lines 55-56.**

Lines 60-62. According to the references provided by the author, I found that the assemblage E have the potential zoonotic transmission, why does the author describe “indicating zoonotic transmission”? Please also check the whole text.

**Response:** **We have revised the sentence in response to the reviewer’s comment. Please see line 54.**

Lines 63-64 and lines73-79. Please add references and citations.

**Response: We have provided the references. Please see lines 56, 66, 67, and 71.**

Line 85 “age group”? In the article, the author only investigated two age groups. Please give an accurate description.

**Response: We agree with the reviewer’s comment. We are sorry for the confusion. We are divided into the three age groups (1−10 days, 11−30 days, and 31−60 days). Our aim is to identify the age group most associated with the occurrence of *Giardia duodenalis*. We have revised this to be precise according to the reviewer’s comment. Please see lines 77-78. An explanation of age group was added to Materials and methods. Please see lines 90-92.**

4. Method.
The calves were housed together? or in a separate cage? Is the sampling season different? Whether seasonal information can be provided? More details about sample collection should be given in the Materials and methods section.

**Response: We understand the reviewer’s concern. Unfortunately, we don’t know the exact information regarding rearing system. The veterinarian collected feces from individual diarrheic calf and sent to us with information such as gender, age, region, and sampling date. We can exactly know the seasonal information by the sampling date. We have provided this information. Please see lines 96-99.**

DNA extraction and PCR. Line 108. The author describes that the SSU rRNA gene locus has highest sensitivity, why not use SSU rRNA genes for screening? I suggest the author re-screen, otherwise the results of the survey may be wrong.

**Response:** **We understand the reviewer’s concern. To be honest, we had not thought about detection of *Giardia duodenalis* using the SSU rRNA gene. This is a big mistake. We used the *bg* gene for the first screening. Most of all, for multilocus analysis, because three loci (*bg*, *gdh*, and *tpi*) are generally used, we did not screen *Giardia duodenalis* using the SSU rRNA gene. We think that analysis of *bg* is appropriate for the detection of *Giardia duodenalis* and in particular, this gives more information regarding the identification of subtype of assemblages.**

5. Discussion is too long and mostly repeats what were described in Results.

**Response:** **We agree with the reviewer’s comment. We have deleted the repetitive sentences as much as possible.**

6. Lines 195-197 and lines 239-240 (In general … animal health.) and (cattle are known …). Please add references and citations.
Lines 266-270. Please add references and citations.

**Response: We have provided the references in response to the reviewer’s comment. Please see lines 249, 290, 323, and 328.**
